# Secretion of S100A8, S100A9, and S100A12 by Neutrophils Involves Reactive Oxygen Species and Potassium Efflux

**DOI:** 10.1155/2015/296149

**Published:** 2015-12-30

**Authors:** Mélanie R. Tardif, Julie Andrea Chapeton-Montes, Alma Posvandzic, Nathalie Pagé, Caroline Gilbert, Philippe A. Tessier

**Affiliations:** Axe de Recherche sur les Maladies Infectieuses et L'immunologie, Centre de Recherche du CHU de Québec-Université Laval, and Département de Microbiologie-Infectiologie et Immunologie, Faculté de Médecine, Université Laval, Québec, QC, Canada G1V 0A6

## Abstract

S100A8/A9 (calprotectin) and S100A12 proinflammatory mediators are found at inflammatory sites and in the serum of patients with inflammatory or autoimmune diseases. These cytoplasmic proteins are secreted by neutrophils at sites of inflammation via alternative secretion pathways of which little is known. This study examined the nature of the stimuli leading to S100A8/A9 and S100A12 secretion as well as the mechanism involved in this alternative secretion pathway. Chemotactic agents, cytokines, and particulate molecules were used to stimulate human neutrophils. MSU crystals, PMA, and H_2_O_2_ induced the release of S100A8, S100A9, and S100A12 homodimers, as well as S100A8/A9 heterodimer. High concentrations of S100A8/A9 and S100A12 were secreted in response to nanoparticles like MSU, silica, TiO_2_, fullerene, and single-wall carbon nanotubes as well as in response to microbe-derived molecules, such as zymosan or HKCA. However, neutrophils exposed to the chemotactic factors fMLP failed to secrete S100A8/A9 or S100A12. Secretion of S100A8/A9 was dependent on the production of reactive oxygen species and required K^+^ exchanges through the ATP-sensitive K^+^ channel. Altogether, these findings suggest that S100A12 and S100A8/A9 are secreted independently either via distinct mechanisms of secretion or following the activation of different signal transduction pathways.

## 1. Introduction

S100A8, S100A9, and S100A12 are small calcium-binding proteins abundantly expressed by neutrophils. S100A8 and S100A9 represent up to 40% of neutrophil cytosolic proteins, whereas close to 5% are S100A12 [1]. These proteins are also expressed by monocytes, macrophages, platelets, and epithelial and endothelial cells following cell stimulation [2–6]. S100A8, S100A9, and S100A12 exist as homodimers, but S100A8 and S100A9 associate in presence of calcium to form the noncovalently bound heterodimer S100A8/A9 (calprotectin).

S100A8, S100A9, and S100A12 induce neutrophil recruitment, adhesion, and release from the bone marrow and are crucial for neutrophil migration to inflammatory sites in response to bacterial infection, LPS, and monosodium urate (MSU) crystals (the causative agent of gout) [7–10]. S100A9 is a potent proinflammatory factor, stimulating neutrophil migration, phagocytosis, and degranulation [9, 11, 12]. S100A12, on the other hand, is a mild activator of granulocyte functions and a potent inducer of mast cell functions [7, 13, 14], and S100A8 is a chemotactic factor for neutrophils [15]. In addition, S100A8 is extremely sensitive to oxidation and nitrosylation, which transforms it into an anti-inflammatory factor [16, 17]. The S100A8/A9 complex has been reported to activate monocyte migration and cytokine secretion [18, 19]. Thus, these damage-associated molecular patterns (DAMPs) control inflammation through distinct but overlapping proinflammatory activities.

S100 proteins lack signal peptides required for the classical Golgi-mediated secretion pathway. Consequently, their release is mediated by alternative secretion pathways [4]. The mechanisms underlying these alternative pathways are unclear, but secretion of S100A8/A9 from monocytes is known to be tubulin-dependent [20]. Moreover, stimulation of neutrophils with MSU crystals leads to the release of S100A8/A9 in a Src kinase-, syk-, and tubulin-dependent manner [8, 10, 21].

High concentrations of S100A8/A9 and S100A12 are found in the serum and at inflammatory sites of patients with acute and chronic inflammation. However, the source of S100A8/A9 and S100A12 remains largely unknown. In some situations, S100A8/A9 release correlates with neutrophil necrosis [22], and this might contribute significantly to the high concentrations found in acute inflammatory lesions or chronic conditions where neutrophil infiltration is significant, such as cystic fibrosis and rheumatoid arthritis [23]. Monocytes secrete S100A9 and S100A8/A9, but not S100A8 alone, upon stimulation with pokeweed mitogen [24]. In addition, GM-CSF, TNF-*α*, IL-1*β*, LPS, and PMA induce the release of S100A8/A9 from monocytes [20, 25]. Likewise, S100A8/A9 was shown to be secreted by neutrophils stimulated with LPS, TNF-*α*, and IL-1*β* [25, 26]. The stress response modulator norepinephrine also induces S100A8 and S100A9 expression in human monocytic cells, suggesting that these proteins may be regulated by stress [27]. Activation of protein kinase C by proinflammatory stimuli and elevation of intracellular [Ca^2+^] following contact with activated endothelium, collagen, or fibronectin can also stimulate S100A8/A9 release from phagocytes [28, 29].

Most of the studies to date have concentrated on the secretion of S100A8/A9, and thus little is known about the mechanisms of secretion of S100A8, S100A9, and S100A12. In order to decipher the type of stimuli triggering the release of S100 proteins from human neutrophils, we exposed neutrophils to a variety of stimuli, including inflammatory mediators, microbes or their derived products, and particulates. Altogether, our results demonstrate that S100A8, S100A9, and S100A8/A9 can be released without the secretion of S100A12 and that the secretion of S100A12 is induced by stimuli promoting the presence of oxidative stress and activation of the NLRP3 inflammasome. The results also demonstrate that the proteins are secreted independently either via distinct mechanisms or following activation of different signal transduction pathways.

## 2. Material and Methods

### 2.1. Ethics Statement

These studies were approved by the CHU de Québec Research Center ethical committee, and all participants gave written informed consent.

### 2.2. Reagents

Triclinic monosodium urate (MSU) crystal preparation was a generous gift from Dr. Paul H. Naccache and generated as described previously [21]. Fullerenes (C_60_) and single-wall carbon nanotubes (60–100 nm in diameter, 1-2 *μ*m in length) were obtained from SES research (Houston, TX, USA). Silica (1.6 *μ*m in diameter) was purchased from US Silica Company (Berkeley Springs, WV, USA). Titanium dioxide (TiO_2_, diameter ≤5 *μ*m), phorbol 12-myristate 13-acetate (PMA), the chemotactic peptide N-formyl-methionyl-leucylphenylalanine (fMLP), H_2_O_2_, protease inhibitors cocktail, sodium orthovanadate (Na_3_VO_4_), and DMSO were purchased from Sigma-Aldrich (St. Louis, MO, USA). IL-8 was obtained from Peprotech Inc. (Rocky Hill, NJ, USA). Leukotriene B_4_ (LTB_4_) and platelet-activating factor (PAF) were purchased from Calbiochem (Gibbstown, NJ, USA). Complement factor 5a (C5a) was obtained from Biovision (Mountain View, CA, USA). Heat-killed* Candida albicans* (HKCA) and Pam3CSK4 were purchased from Invivogen (San Diego, CA, USA). Unpurified autologous decomplemented serum was used as a source of opsonins when indicated. Serum was heat-inactivated for 30 min at 56°C and stored at −20°C. The zymosan, HKCA, or MSU suspensions were opsonized by incubation with 50% autologous serum for 30 minutes at 37°C before being washed and resuspended in Hank's balanced saline solution (HBSS). The monoclonal antibody 27E10 was purchased from HyCult Biotechnology (Canton, MA, USA). The HRP-conjugated antibodies were purchased from Jackson ImmunoResearch. The cOmplete Ultra protease inhibitor cocktail was obtained from Roche (Laval, Canada).

### 2.3. Production and Purification of Recombinant Proteins

Cloning, expression, and purification of human S100A8, S100A9, and S100A12 were described previously [8]. Endotoxins were removed from the protein solutions using Acticlean Etox column (Sterogene Bioseparation). Contamination by endotoxins was always less than 1 pg of LPS/*μ*g of recombinant proteins as measured by the* Limulus* amebocyte assay (Lonza). The proteins were kept at −80°C for up to 6 months until used.

### 2.4. Sandwich ELISA

High-binding 96-well plates were incubated overnight at 4°C with 100 *μ*L of a solution of mAbs clone 1F8 (anti-S100A8; 0.5 *μ*g/mL), 6B4 (anti-S100A9; 2.5 *μ*g/mL), 2A10 (anti-S100A12; 1 *μ*g/mL), or the polyclonal anti-S100A9 (2.5 *μ*g/mL) diluted in 0.1 M carbonate buffer (pH 9.6). The plates were washed three times with PBS/0.1% Tween and the nonspecific binding sites were blocked by the addition of PBS/0.1% Tween/2%BSA for 45 min at room temperature. One hundred *μ*L of samples (diluted in blocking buffer) and recombinant proteins or purified calprotectin (used as standard curve) diluted in blocking buffer was then added. The plates were extensively washed 45 min later and 100 *μ*L of the polyclonal anti-S100A8 (4 *μ*g/mL), anti-S100A9 (0.5 *μ*g/mL), anti-S100A12 (0.25 *μ*g/mL), or the monoclonal anti-calprotectin clone 27E10 (0.075 *μ*g/mL) diluted in blocking buffer was added. After 45 min, the wells were washed three times and 100 *μ*L of HRP-conjugated anti-rabbit or mouse IgGs was added to the wells for 45 min. The wells were washed three times and TMB substrate was added. The reaction was stopped by the addition of H_2_SO_4_ 0.18 M and the optical density was read at 450 nm.

### 2.5. Isolation of Human Neutrophils

Peripheral blood was collected in heparinized tubes from healthy adult volunteers and neutrophils were isolated as described previously [21]. Cells were resuspended in HBSS1x containing physiological concentrations of Ca^2+^ (1.3 mM) and Mg^2+^ (0.8 mM) and 10 mM HEPES, pH 7.4. The purity and cell viability of neutrophils preparations were always ≥98% as assessed by acetic blue staining and trypan blue exclusion, respectively.

### 2.6. Isolation and Purification of Calprotectin from Human Neutrophils

Calprotectin was purified from human neutrophils as described previously [21]. The purity of calprotectin was confirmed by SDS-PAGE under nonreducing conditions. Less than 20% of the S100A8 and S100A9 were in homodimer forms. The calprotectin was aliquoted and kept in −20°C until used.

### 2.7. Stimulation of Human Neutrophil

Neutrophils (10^6^ cell/mL in a final volume of 500 *μ*L) were preincubated with protease inhibitors to avoid proteolysis for 5 min and then incubated in presence or absence of stimuli like LTB4 (50 nM), C5a (100 nM), PAF (10 *μ*M), IL-8 (10 ng/mL), MSU crystals (1 mg/mL), PMA (10 nM), fMLP (1 *μ*M), TNF (50 ng/mL), GM-CSF (20 nM), TiO_2_ (1.5 mg/mL), silica (3 mg/mL), single-wall carbon nanotubes (1.5 mg/mL), fullerenes (1.5 mg/mL), heat-killed* C. albicans* (10^8^ cells/mL), zymosan (1 mg/mL, i.e., 60 × 10^6^ particles/mL), Pam3Csk4 (10 *μ*g/mL), H_2_O_2_ (250 *μ*M), or their respective vehicle (HBSS1X or DMSO (<0.1%)) for 60 min at 37°C under gentle agitation (300 rpm). Stimulations were stopped by centrifugation at 1,500 ×g for 15 s, and the supernatants were harvested and frozen at −20°C until analysed. Cell viability was determined at the end of the stimulation by trypan blue staining. Crude neutrophil extracts were obtained by lysis of 40 × 10^6^ cells in 400 *μ*L of RIPA lysis buffer (Tris 50 mM pH 8.0, 150 mM NaCl, 0.1% SDS, 1% NP-40, and Roche protease inhibitor cocktail). Samples were kept at −20°C until used.

### 2.8. Sequential Solubilisation of Intracellular Compartments from Human Neutrophils

Neutrophil pellets recovered after stimulation were solubilised with 500 *μ*L of hypotonic buffer (20 mM Tris-HCl pH 7.5, 10 mM NaCl, 1 mM EDTA, 0.1% NP-40, protease inhibitors, and 2 mM Na_3_VO_4_), kept on ice for 30 min, and centrifuged at 13,000 ×g for 10 min at 4°C. The soluble fractions, which contained cytosolic proteins, were transferred to fresh tubes and frozen at −20°C. The pellets were dissolved in 500 *μ*L of hypertonic buffer (20 mM Tris-HCl pH 7.5, 400 mM NaCl, 1 mM EDTA, 1% NP-40, 0.1% SDS, protease inhibitors, 2 mM Na_3_VO_4_) known to solubilise membrane-bound proteins and centrifuged at 13,000 ×g for 10 min at 4°C. The soluble material of this second solubilisation step was transferred into fresh tubes and frozen at −20°C. The pellets were next dissolved into 500 *μ*L of 10 mM Tris-HCl pH 7.5, 100 mM NaCl, 1 mM EDTA, 10% glycerol, 1% Triton X-100, 0.1% SDS, protease inhibitors, 2 mM Na_3_VO_4_ to solubilise cytoskeleton and lipid raft-bound proteins and kept frozen at −20°C until used for ELISA and SDS-PAGE/western blotting under nonreductive conditions.

### 2.9. Isolation of Microvesicles (MV)

PMNs were stimulated with 1.5 mg/mL of the particulate agonists MSU crystal for 60 min at 37°C. Cell activation was stopped by centrifugation (10 min, 400 ×g at 4°C) and supernatants were collected. Supernatants (S1) were then centrifuged at 10,000 ×g/10 min and the supernatant and pellet (S2, P2) were collected. MV from PMNs were concentrated by ultracentrifugation for 45 min at 100,000 ×g (S3, P3), and the resulting supernatant (S3) was then centrifugated again for 60 min at 160,000 ×g (S4, P4) at 4°C. Calprotectin secretion was estimated by measuring the protein in the different fractions by ELISA. Immunoblotting of proteins S100A8 and S100A9 in the fractions with MV of neutrophils treated with MSU or HBSS was performed.

### 2.10. Proteomic Analysis

Neutrophils from 10 different blood donors were stimulated with fMLP (10^−7^ M), MSU crystals (1.5 mg/mL), fullerenes (1.5 mg/mL), or their diluents for 30 min at 37°C. Equal volumes of supernatants were pooled, and then 1 *μ*g of recombinant papaya mosaic virus core protein (internal control, generous gift from Denis Leclerc, Université Laval) was added to the pooled samples (as an internal standard). One hundred *μ*g of the pooled supernatants was precipitated overnight with 5 vol of acetone and resuspended in triethylammonium bicarbonate 0.5 M containing 0.5% sodium deoxycholate. Proteins were then reduced and alkylated according to the isobaric tags for relative and absolute quantitation (iTRAQ) kit manufacturer's instructions (AB SCIEX). Samples were digested with trypsin (Sequence grade Modified, Promega) using 1 : 30 ratio overnight at 37°C. After digestion, peptides were acidified to precipitate sodium deoxycholate and then purified with an oasis HLB cartridge (1 cc, 10 mg, Water Corp.) and lyophilized. Dried peptides were dissolved in 30 *μ*L 0.5 M triethylammonium bicarbonate 0.5 M and labeled with iTRAQ label reagent (AB SCIEX). 4-plex labeling was performed for 2 h at room temperature in the dark. Labeled peptides were combined in one tube and dried with the SpeedVac. Samples were cleaned up using HLB cartridge (Water Corp.). Samples were dried and reconstituted in 200 *μ*L HPLC water and 1/100 ampholytes pH 3–10 (Biorad). The peptides were then fractionated on two 18 cm immobilized pH gradient strips pH 3–6 and pH 3–10 using isoelectric focusing. Immobilized pH gradient strips were passively rehydrated 5 hours. Focusing was performed according to the following protocol: 0–250 V (gradient over 15 min); 250–4000 V (gradient over 2 h); 4000 V (fixed, until a total of 10000 Vh). Strips were cut in 72 fractions and peptides were extracted in 2% ACN, 1%FA solution followed by 50% ACN, 1% FA. Finally, fractions were dried with the SpeedVac.

The MS analysis was performed on a QSTAR XL (AB SCIEX, Foster City, CA) in an information-dependent acquisition (IDA) mode. Mass spectra were acquired across 400–1600* m/z* for 1 s followed by 3 MS/MS of 3 s per cycle. A dynamic exclusion window of 15 s was used. Rolling collision energy was on with CE adjustment for iTRAQ reagent analysis.

Protein/peptide identification and quantification were performed using Protein Pilot Software version 3.0 searching a Uniprot database containing human proteins and common contaminants. Only proteins identified by at least three peptides and with a *P* value less than 0.05 were used for quantitation. Relative concentrations of the 123 proteins identified in the supernatants were then normalized according to the relative concentration of the internal control (papaya mosaic virus core protein).

### 2.11. Statistical Analyses

All experiments were performed three to six times using neutrophils from different donors. The results are expressed as mean ± SEM of separate experiments. Statistical analyses were performed using Bonferroni multiple comparison test except when stated in figure legend. The results were considered significant if *P* values were less than 0.05. All statistical analyses were performed using GraphPad Prism 5.0 (GraphPad Software Inc., San Diego, CA, USA).

## 3. Results

### 3.1. Neutrophils Exposed to Stress Release S100A8, S100A9, Calprotectin, and S100A12

**Figure 1:**
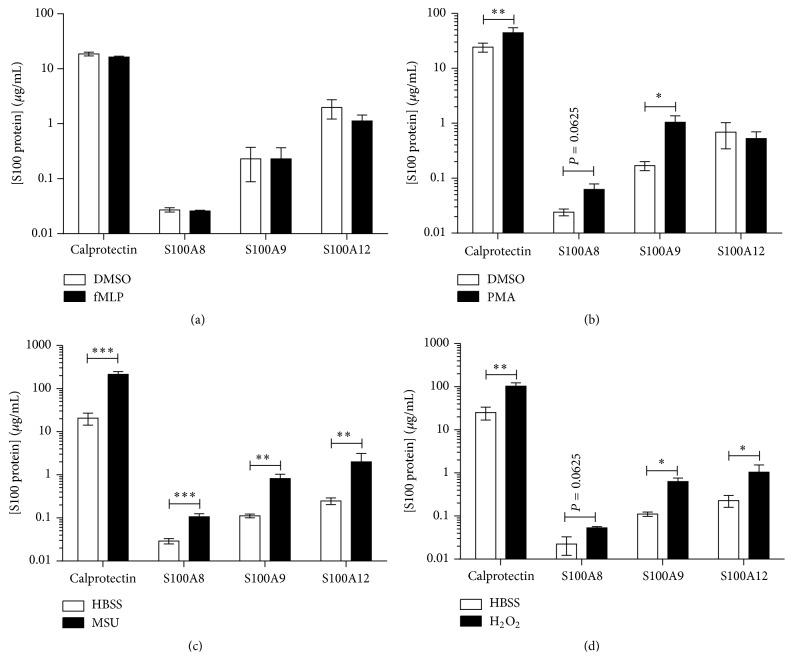
Secretion of S100A8, S100A9, S100A12, and S100A8/A9 by human neutrophils. Neutrophils were stimulated with (a) 10^−7 ^M fMLP, (b) 10 nM PMA, (c) 250 *μ*M H_2_O_2_, or (d) 1.5 mg/mL MSU crystals for 1 h as described in Section 2. Cells were then centrifuged and supernatants were harvested to perform ELISA for S100A8, S100A9, S100A12, or S100A8/A9. Results represent the means ± SEM of 4 donors. ^*∗*^ *P* < 0.05; ^*∗∗*^ *P* < 0.01; ^*∗∗∗*^ *P* < 0.001.

Using specific ELISA described in Supplementary Data, in Supplementary Material available online at http://dx.doi.org/10.1155/2015/296149 (Supplementary Figures  1–3), we investigated the secretion of S100A8, S100A9, and S100A12 by neutrophils. Neutrophils were stimulated with fMLP, MSU, PMA, or H_2_O_2_ to study the secretion of S100A8, S100A9, and S100A12. fMLP, a powerful activator of neutrophils triggering degranulation, failed to induce the secretion of calprotectin, S100A8, S100A9, or S100A12 (Figure 1(a)). Other chemotactic agents tested, including C5a, PAF, IL-8, and LTB_4_, also failed to induce the release of calprotectin and S100A12 (Supplementary Figure 4). Absence of S100 proteins in the supernatants was not due to degradation by proteases as addition of protease inhibitors only modestly increased detected concentrations in unstimulated and stimulated supernatants. PMA, which has been reported to promote the secretion of calprotectin from human monocytes, weakly stimulated the secretion of calprotectin (1.8-fold), S100A9 (7-fold), and S100A8 (2.6-fold) (although the latter was not significant), but not consistently of S100A12 from human neutrophils (Figure 1(b)). Similarly, cells exposed to H_2_O_2_, which mimics oxidative stress found in inflammatory environment, released calprotectin, S100A9, S100A8, and S100A12 with 7-, 5.4-, 2.4-, and 5.6-fold increases, respectively (Figure 1(c)). MSU crystals, which we previously reported to induce the release S100A8/A9 [21], led to S100A8, S100A9, and S100A12 secretion from neutrophils with 3.6-, 7-, and 8-fold increases, respectively, compared to vehicle (Figure 1(d)). These results indicate that S100A8 and S100A9 can be secreted independently of each other by neutrophils, and not exclusively in a heterodimer form (calprotectin). As S100A8 and S100A9 homodimers were secreted together with calprotectin, we focused the rest of our studies on S100A8/A9 and S100A12.

### 3.2. GM-CSF Induces Secretion of Calprotectin and S100A12

**Figure 2:**
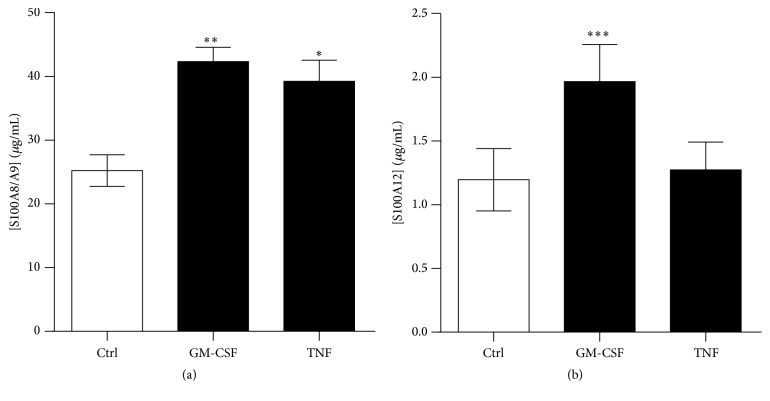
Secretion of calgranulins by neutrophils stimulated with GM-CSF and TNF. Neutrophils were stimulated with 20 nM GM-CSF or 50 ng/mL TNF for 60 minutes. (a) S100A8/A9 and (b) S100A12 were then quantified by ELISA. Results represent the means ± SEM of 5 donors. ^*∗*^ *P* < 0.05; ^*∗∗*^ *P* < 0.01; ^*∗∗∗*^ *P* < 0.001.

Various neutrophil functions, such as adhesion, superoxide release, and phagocytosis, are known to be activated or potentiated by hematopoietic growth factors or inflammatory cytokines, including GM-CSF and TNF-*α*. These cytokines may contribute not only to host defense against invading microorganisms but also to tissue damage at inflammatory sites [30]. Stimulation of neutrophils with TNF-*α* or GM-CSF led to 1.56- and 1.68-fold increases in the secretion of S100A8/A9 (Figure 2). TNF-*α* did not induce secretion of S100A12, while GM-CSF led to a 1.64-fold increase in its release.

### 3.3. Dectin-1 Ligand Stimulates the Release of S100A8/A9 and S100A12

**Figure 3:**
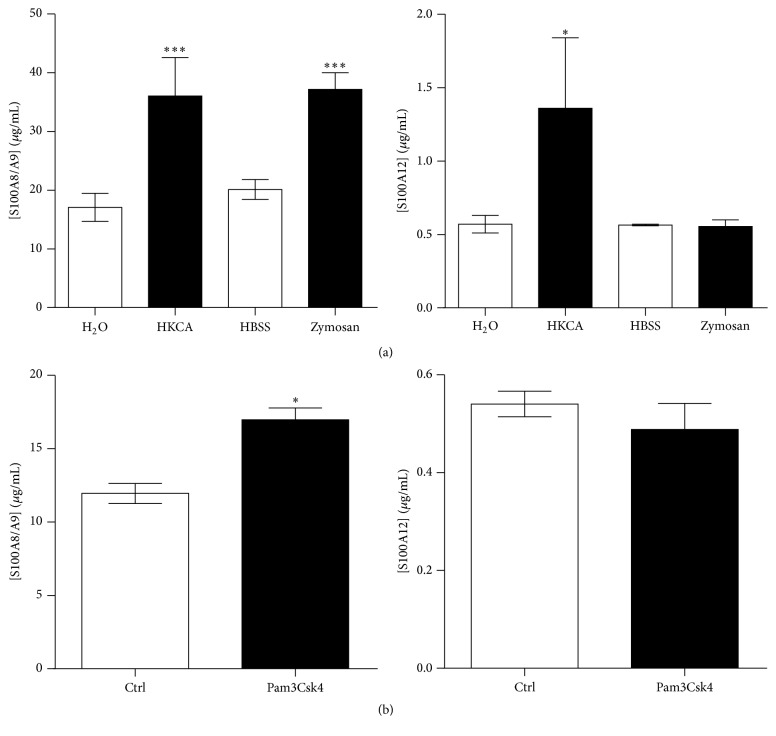
Effects of microbe-derived products and whole yeasts on the release of S100A8/A9 and S100A12. (a)* C. albicans* (10^8^ cells/mL) enhances the secretion of S100A8/A9 and S100A12, whereas zymosan (1.5 mg/mL) induces only the release of S100A8/A9. (b)* Pam3CSK4* (TLR2 ligand, 10 *μ*g/mL) potentiates the release of S100A8/A9 but not S100A12. Neutrophils were incubated with whole microorganisms or microbe-derived product for 60 min at 37°C. The concentrations of secreted S100A8/A9 and S100A12 were determined by ELISA. Results are the mean ± SEM of at least three independent experiments performed on neutrophils from different donors. ^*∗*^ *P* < 0.05 and ^*∗∗*^ *P* < 0.01 compared with control by paired *t*-test.

After recruitment to the site of infection, neutrophils first detect microbes through different pathogen-recognizing receptors (PRR), including Toll-like receptors (TLR) and C-type lectins, such as dectin-1. Because S100A8/A9 and S100A12 exhibit antimicrobial proprieties, particularly against yeast and fungi [22, 25, 26], we investigated the effect of microbe-derived products or whole microorganisms on the secretion of these proteins. For this purpose, neutrophils were incubated with Pam3CSK4 (TLR2 ligand), zymosan (yeast cell wall, TLR2/dectin-1 ligand), and heat-killed* C. albicans* (HKCA; dectin-1 ligand). Stimulation with HKCA led to the secretion of S100A8/A9 and S100A12 (Figure 3). In contrast, zymosan or the TLR2 ligand Pam3CSK4 induced the secretion of S100A8/A9 but not S100A12. Thus, S100A8/A9 secretion is increased in response to activation of TLR2 and dectin-1 signaling by pathogen-associated molecular patterns (PAMPs), but only a full activation of dectin-1 induces the release of S100A12.

### 3.4. Particulates Stimulate the Secretion of S100A8/A9 and S100A12 by Neutrophils

**Figure 4:**
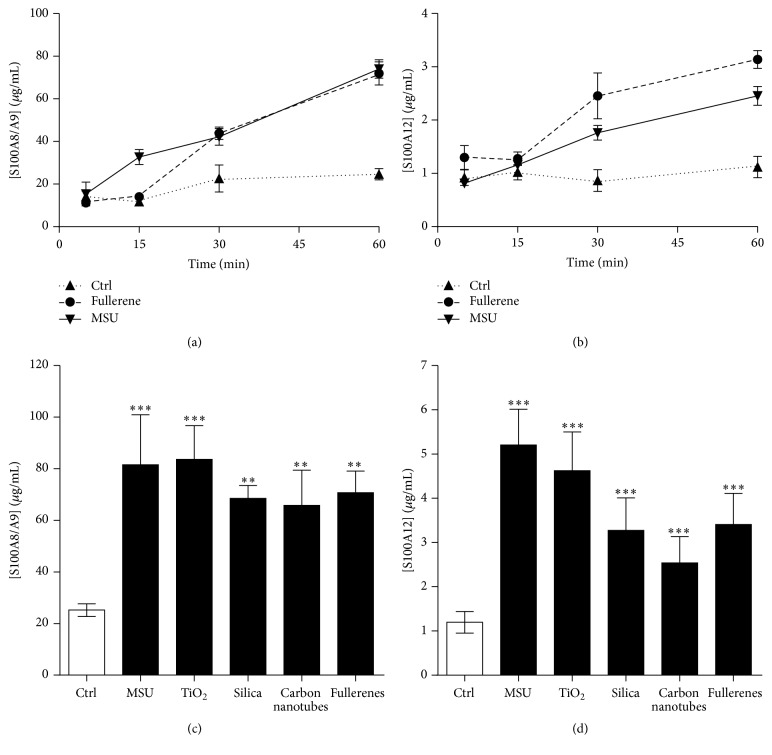
Phagocytic particles induce the secretion of S100A8/A9 and S100A12. Neutrophils were stimulated with 1.5 mg/mL MSU crystals or fullerenes for increasing periods of time. (a) S100A8/A9 and (b) S100A12 in the supernatants were then quantified by ELISA. (c and d) Neutrophils were stimulated with 1.5 mg/mL MSU crystals, 1.5 mg/mL TiO_2_, 3 mg/mL silica, 1.5 mg/mL carbon nanotubes, or 1.5 mg/mL fullerenes for 60 minutes. (c) S100A8/A9 and (d) S100A12 in the supernatants were then quantified by ELISA. Results represent the means ± SEM of 5 donors. ^*∗∗*^ *P* < 0.01; ^*∗∗∗*^ *P* < 0.001.

Of all the stimuli tested, MSU crystals were the most powerful trigger of S100 proteins secretion. We hypothesized that their spindle-like shape could participate in neutrophil stimulation. Therefore, we examined whether other agonists of similar shape also stimulated the secretion of S100A8/A9 and S100A12. Neutrophils were exposed to MSU crystals (1–40 *μ*m), silica (1.6 *μ*m), TiO_2_ (<5 *μ*m), or single-wall carbon nanotube (100 nm in diameter; 1-2 *μ*m in length), and these agonists share a common spindle-like structure. Fullerenes were used as controls because they have the same chemical composition as carbon nanotubes (i.e., pure carbon) but in a spherical conformation. All particulate agonists induced the secretion of S100A8/A9 and S100A12, MSU and TiO_2_ being the most efficient at promoting secretion (Figure 4). Taken together, these results indicate that the nature of the stimulus, but not its shape, determines the release of S100A8, S100A9, calprotectin, and S100A12 by neutrophils.

### 3.5. Role of Redox in the Secretion of Calprotectin and S100A12

**Figure 5:**
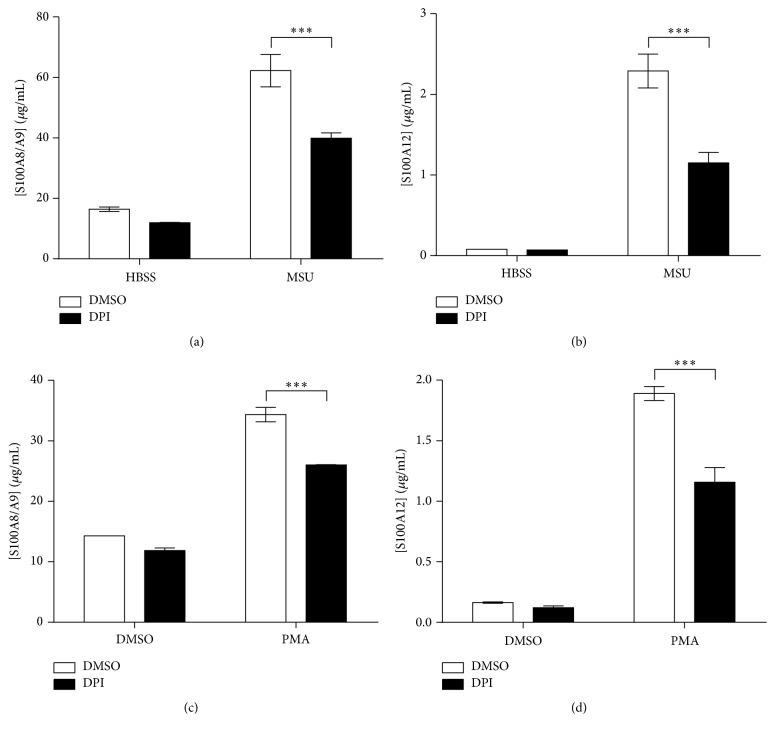
Inhibition of calgranulin secretion by DPI. Neutrophils were preincubated with the NADPH oxidase inhibitor DPI (10 *μ*M) and then stimulated with 1.5 mg/mL MSU crystals or 10 nM PMA for 60 minutes. (a and b) S100A8/A9 and (c and d) S100A12 in the supernatants were then quantified by ELISA. Results represent the means ± SEM of 5 donors. ^*∗∗∗*^ *P* < 0.001.

Damage associated-molecular patterns (PAMPs) like MSU crystals trigger the alternative secretion of IL-1*β* through the activation of inflammasome and caspase-1, a phenomenon linked to K^+^ efflux and ROS production [31]. As MSU crystals and H_2_O_2_ induced the release of S100A8/A9 and S100A12, we examined the role of redox in the secretion of S100 proteins. Neutrophils were stimulated with MSU crystals or PMA in presence of the NADPH oxidase inhibitor diphenyleneiodonium sulfate (DPI) (Figure 5). As expected, DPI reduced ROS production in neutrophils (Supplementary Figure 5). DPI mildly reduced the secretion of S100A8/A9 and S100A12 by unstimulated cells. In contrast, DPI inhibited S100A8/A9 and S100A12 secretion by 36% and 50%, respectively, when neutrophils were stimulated with MSU (Figures 5(a) and 5(b)). Similarly, DPI decreased S100A8/A9 and S100A12 secretion by neutrophils stimulated with PMA (Figures 5(c) and 5(d)). Altogether, the results suggest that redox status plays a role in the secretion of both S100A8/A9 and S100A12 as it is the case for IL-1*β* in human monocytes/macrophages.

### 3.6. K^+^ Efflux from ATP-Sensitive K^+^ Channels Is Involved in S100A8/A9 and S100A12 Secretion

**Figure 6:**
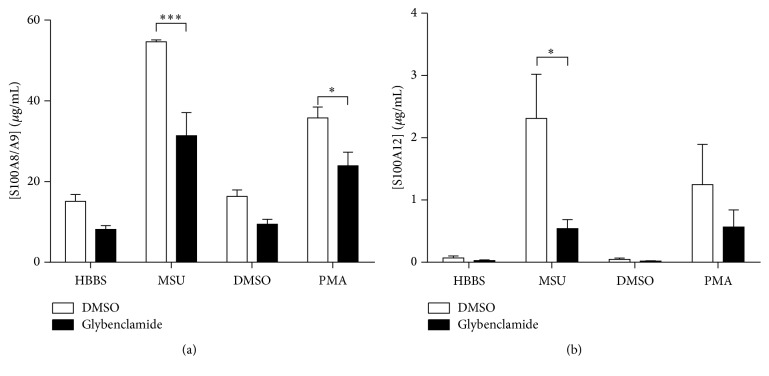
Inhibition of calgranulin secretion by glibenclamide. Neutrophils were preincubated with the ATP-sensitive K^+^ channel inhibitor glibenclamide (50 *μ*M) and then stimulated with 1.5 mg/mL MSU crystals or 10 nM PMA for 60 minutes. (a) S100A8/A9 and (b) S100A12 in the supernatants were then quantified by ELISA. Results represent the means ± SEM of 5 donors. ^*∗*^ *P* < 0.05; ^*∗∗∗*^ *P* < 0.001.

The role of K^+^ efflux from ATP-sensitive K^+^ channels in the secretion of S100A8/A9 and/or S100A12 by neutrophils exposed to inflammatory conditions was investigated as it regulates the alternative secretion of IL-1*β* by macrophages stimulated with LPS and ATP [32–34]. Neutrophils were treated with glibenclamide or DMSO (vehicle control) before the addition of MSU. Preincubation of cells with glibenclamide reduced the secretion of S100A8/A9 by MSU-stimulated neutrophils by 42% compared to cells pretreated with DMSO (Figure 6). A similar inhibition (44%) was observed in unstimulated cells. Secretion of S100A12 was reduced by 64% and 73%, respectively, in unstimulated and MSU crystal-stimulated neutrophils. Similar inhibition was observed when cells were stimulated with PMA. Glibenclamide had no apparent cytotoxic effect on neutrophils during the course of stimulation (data not shown). To confirm the effect of glibenclamide, neutrophils were incubated in medium containing high K^+^ concentrations. As with glibenclamide, inhibition of the K^+^ efflux led to a reduction of S100A8/A9 secretion induced by MSU crystals (Supplementary Figure 6). Neutrophils were then incubated with ouabain, an inhibitor of the activity of the Na^+^/K^+^ ATPase. Inhibition of the Na^+^/K^+^ ATPase by ouabain had no effect on S100A8/A9 and S100A12 secretion from neutrophils exposed or not to MSU or PMA (data not shown). These results suggest that K^+^ exchange through the ATP-sensitive K^+^ channels participates in the stimulated secretion of S100A8/A9 and S100A12, as is the case for IL-1*β* in monocytes/macrophages.

### 3.7. S100A8 and S100A9 Move toward Cytoskeleton/Lipid Rafts Fractions upon Neutrophils Activation by MSU Crystals

**Figure 7:**
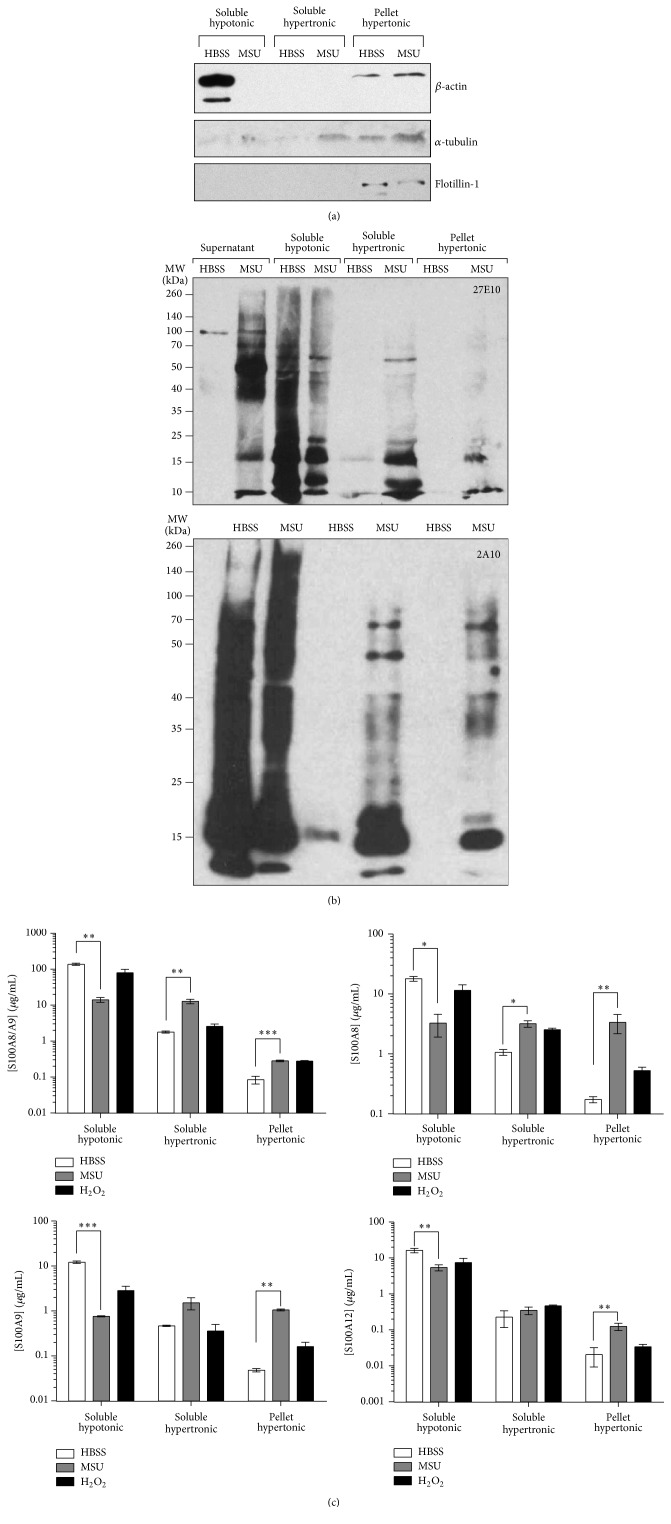
S100A8, S100A9, S100A12, and calprotectin move toward cytoskeleton/lipid raft fractions following neutrophils stimulation by H_2_O_2_ or MSU. Neutrophils were stimulated with 1.5 mg/mL MSU crystals or 250 *μ*M H_2_O_2_ and supernatants harvested before sequential cells lysis as described in Section 2. Fractions were loaded onto SDS-PAGE and (a) *β* actin, *α* tubulin, and flotillin-1 or (b) S100A8/A9 or S100A12 was detected by immunoblotting. Results are from one experiment are representative of 2 others. (c) Fractions were subjected to sandwiches ELISA against S100A8, S100A9, S100A12, or S100A8/A9. Results represent the mean ± SEM of 4 donors quantified in duplicate. ^*∗*^ *P* < 0.05; ^*∗∗*^ *P* < 0.01; ^*∗∗∗*^ *P* < 0.001, MSU versus HBSS.

Calprotectin translocates into tubulin-enriched compartments in PMA-stimulated monocytes [20], a phenomenon preceding its release. Considering that we found a significant secretion of calprotectin, S100A8, S100A9, and S100A12 we expected that similar phenomenon occurred in neutrophils. To confirm this hypothesis, neutrophils were subjected to sequential lysis in buffers of increasing tonicity to determine the forms and localization of calprotectin, S100A8, S100A9, and S100A12. Beta actin, alpha tubulin, and lipid raft marker flotillin were used as markers of cytosol, cytoskeleton, and membranes (Figure 7(a)). The majority of calprotectin was detected in dimer forms, mainly in the cytosol fraction (soluble hypotonic) of resting neutrophils (Figure 7(b)). However, calprotectin was also retained in the stacking gels of soluble hypotonic fractions of resting neutrophils (data not shown) suggesting the presence of heterooligomeric forms in unstimulated neutrophils. Interestingly, the larger forms of calprotectin within cytoplasm decreased following stimulation with MSU crystals. This could be related to the massive secretion of the heterocomplex forms. Moreover, the heterodimers, and to a lesser extent the high molecular weight heterocomplex forms, moved toward soluble and insoluble hypertonic fractions (membrane and cytoskeleton/lipid raft compartments) in neutrophils exposed to MSU. Thus, stimulation of neutrophils with MSU crystals leads to the redistribution of high molecular weight forms of calprotectin from the cytosol to the membranes and lipid rafts, leading to their secretion.

The different forms of S100A proteins were next quantified in each fraction by ELISA to confirm their localization. Homomeric forms of S100A8 and S100A9 were detected in the cytosol of neutrophils (approximately 10 times less S100A8, S100A9, and S100A12 than calprotectin, Figure 7(c)). As expected, the subcellular localization of the S100A proteins changed following neutrophil stimulation and the proteins moved toward membrane and cytoskeleton/lipid raft fractions. This phenomenon was more pronounced for S100A8 and S100A9 when neutrophils were exposed to MSU and to a lesser extent H_2_O_2_ (18x and 2x increases, resp., for S100A8 and 22x and 3x for S100A9). Similar trends were observed for S100A12 (with a 12-fold increase following MSU stimulation) but fewer proteins were associated with cytoskeleton and/or lipid rafts compared to S100A8 and S100A9. Relocalization of calprotectin to cytoskeleton/lipid rafts fraction was not as marked (with 3-fold increase). Altogether, these results indicate that calprotectin, S100A8, S100A9, and S100A12 localize to cytoskeleton/lipid rafts in neutrophils following activation.

### 3.8. S100 Proteins Are Not Secreted through Degranulation or Netosis

S100A8 and S100A9 have been reported in gelatinase and specific granules while S100A12 and S100A8/A9 were reported in specific granules [35]. Others have reported S100A8/A9 secretion through netosis [36]. To decipher the mechanism of secretion, we compared relative protein concentrations in supernatants from neutrophils stimulated with MSU crystals and fullerenes (both inducing degranulation and the release of S100A8/A9 and S100A12) with those of neutrophils stimulated with fMLP (inducing degranulation, but not the secretion of S100A8/A9) to confirm that secretion of S100A8/A9 was not linked to degranulation. A 30 min stimulation was also chosen as we could detect secretion of S100A8/A9 at this early time point, but not netosis by microscopy (data not shown). Concentrations of catalase were slightly increased in MSU- (2.5-fold) and fullerene-stimulated (1.1-fold) supernatants (Table 1). However, concentrations of histone 1 and 2B were similar or slightly lower in MSU and fullerene-stimulated supernatants, compared to supernatants of neutrophils stimulated with fMLP. Thus, we found no relationship between relative concentrations of the netosis associated proteins catalase and histones 1 and 2 and secretion of S100A8, S100A9, and S100A12, suggesting that netosis may not be involved as a mechanism of secretion. In addition, concentrations of antimicrobial proteins and enzymes like metalloproteinase-9, elastase, and lactoferroxin C (found in granules) were increased in MSU-stimulated supernatants compared to fMLP (ratios of 4.5, 4.5, and 5.7, resp.), but not in fullerenes-stimulated supernatants (Table 1), indicating no correlation between degranulation and secretion of S100A8/A9 or S100A12. These results were confirmed by examining PMN degranulation induced by MSU crystals or fMLP in absence or presence of cytochalasin B, known to potentiate neutrophil degranulation [37]. Complete degranulation induced by fMLP and cytochalasin B did not increase calprotectin (Supplementary Figure 7) and S100A12 secretion. Relative concentrations of lactate dehydrogenase, myosin, and beta actin, which were not increased in MSU or fullerene-stimulated supernatants, confirmed that secretion of S100A8, S100A9, and S100A12 was not due to cell leakage (Table 1). Together, these results strongly suggest that S100A8/A9 and S100A12 are not secreted by degranulation, netosis, or cell necrosis.

### 3.9. Calprotectin and S100A12 Are Mainly Secreted in a Nonvesicular Form

**Figure 8:**
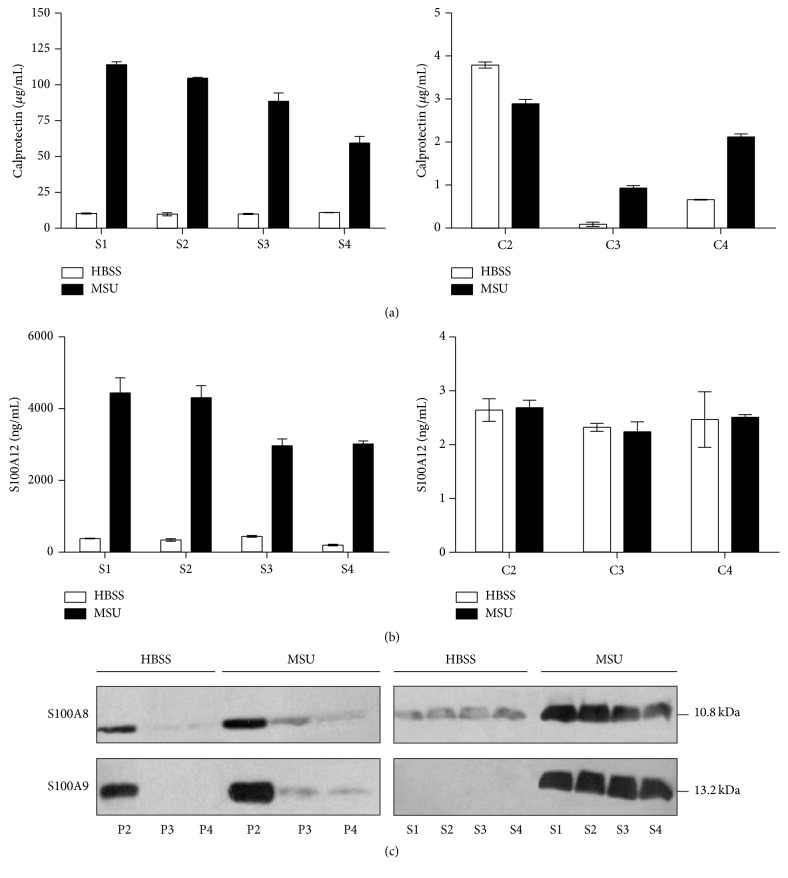
S100A8/A9 and S100A12 are almost absent from microvesicles. Neutrophils were incubated with MSU crystals (1.5 mg/mL) or its diluent for 60 min at 37°C. The MV were isolated after sequential centrifugation. The concentrations of (a) S100A8/A9 and (b) S100A12 were determined by ELISA in the supernatant fractions (S1, S2, S3, and S4) and the pellet fractions (P1, P2, and P3) containing the MV. Data are the mean ± SEM of three separate experiments. (c) The presence of S100A8 and S100A9 in the supernatants and MV was determined by western blot. Results are from one experiment representative of 2 others.

Alternative secretion occurs via vesicular and nonvesicular pathways. We therefore investigated whether calprotectin and S100A12 were secreted in a vesicular-dependent manner from neutrophils stimulated with MSU. Microvesicles (MV) were isolated after sequential centrifugation from the supernatants of neutrophils stimulated with MSU crystals. Microvesicles were separated in large (P2), intermediate (P3), and small microvesicles (ectosomes, P4) by sequential centrifugation. Calprotectin and S100A12 were mostly found in the soluble fractions (Figures 8(a) and 8(b), S1 to S4). Almost no S100A12 was detected in vesicular fractions (P2 to P4). However, some calprotectin was detected in the large microvesicles in unstimulated and stimulated PMN and its presence in smaller vesicles was enhanced by cell stimulation. Disruption of vesicular membranes by adding 0.5% triton did not improve the detection of S100A8/A9 or S100A12, confirming that most of the S100 proteins found in the supernatant were not trapped into microvesicles. These results were confirmed by western blot (Figure 8(c)).

## 4. Discussion

The secretion of proteins through alternative pathways is a complex process involving multiple signals and protein-protein interactions. These interactions differ according to the cell type and the nature of the stimulus. In this study, we examined the secretion of 4 related proteins (S100A8/A9, S100A8, S100A9, and S100A12) from freshly isolated human neutrophils in response to inflammatory stimuli. Stimulation with MSU crystals and other phagocytic particles increased the secretion of all calgranulins while secretion was restricted to S100A8/A9, S100A8, and S100A9 when cells were activated by PMA. In contrast, chemotactic factors like fMLP, C5a, and IL-8 did not induce the secretion of calgranulins. Activation of neutrophils led to the translocation of heterodimers (S100A8/A9) and homodimers (S100A8, S100A9, and S100A12) from the cytosol to the cytoskeleton and membrane before secretion. Similar to IL-1*β* secretion, the alternative secretion pathway used by calgranulins was dependent on ROS production and K^+^ fluxes. The secretion was not associated with netosis or degranulation, and the majority of secreted calgranulin was found in soluble form, but some was associated with large vesicles. Thus, stimulation of neutrophils leads to the translocation of calgranulins to the cytoskeleton/membrane before their secretion.

S100A8/A9, S100A8, S100A9, and S100A12 have been found at inflammatory sites and in the serum in acute and chronic inflammation [38], and several reports indicate that they exert proinflammatory activities and are involved in phagocyte migration. S100A8, S100A9, and S100A8/A9 have been associated with granulocyte and monocyte adhesion to endothelium, as well as with their migration across endothelial cells [8, 28, 39, 40]. In addition, a report suggests that S100A8/A9 promotes the expression of adhesion molecules such as ICAM-1 and VCAM-1 and downregulates the expression of tight junction proteins on endothelial cells [41]. S100A12, on the other hand, stimulates granulocyte adhesion [7]. More recently, Wei et al. described mast cell and monocyte recruitment induced by S100A12 [13], while Yang et al. described mast cell degranulation induced by S100A12 [14]. S100A8/A9 and S100A12 have also been reported to inhibit microbial growth [42–44]. These data suggest distinct biological activities for these proteins, which should be reflected in their patterns of secretion. Indeed, we found that stimuli usually found near or on endothelium predominantly favored the secretion of S100A8/A9 over that of S100A12. For example, TNF*α* and PAF (which are produced by or near endothelial cells during inflammatory reactions) induced the secretion of S100A8/A9 but had almost no effect on S100A12 secretion. This contrasts with the myeloid growth factor GM-CSF, which promotes the release of both S100A8/A9 and S100A12. This suggests that stimuli found near endothelium induce the release of S100A8/A9, which could then promote phagocyte migration through the expression of adhesion molecules and downregulation of tight junction proteins by endothelial cells [41]. Interestingly, secretion of S100A8/A9 was always associated with the secretion of S100A8 and S100A9 homodimers.

Most of S100A8 and S100A9 were secreted in the heteromeric form (calprotectin) by resting and stimulated neutrophils. As reported by others [45], western blot analyses in nonreductive conditions suggested that the major forms of secreted S100A8/A9 were heterotetramers and, to a lesser extent, heterooctamers followed by heterodimers (data not shown). Interestingly, monomeric/homocomplex forms of S100A9 were secreted by neutrophils stimulated with H_2_O_2_, MSU crystals, and PMA with concentrations reaching 0.5 to 2 *μ*g/mL whereas very little S100A8 was released, only in response to MSU crystals (approximately 100 ng/mL). Nonetheless, such concentrations are sufficient to induce chemotaxis and cytokine secretion in response to S100A8 and S100A9 [9]. The secretion of S100A12 by neutrophils was at least 20-fold inferior to S100A8/A9 (0.5 to 5 *μ*g/mL) and was significantly triggered once cells were exposed to MSU crystals or H_2_O_2_, but not PMA.

S100A8/A9 and S100A12 have been reported in granules [35], suggesting that they could be released following neutrophil degranulation. In addition, Hetland et al. previously reported the release of S100A8/A9 by neutrophils stimulated with fMLP [46], a potent inducer of degranulation. However, the main conclusion of this study was based on the disappearance of intracellular S100A8/A9 after stimulation, and the authors of the study were not able to detect S100A8/A9 in the supernatants of stimulated neutrophils. We did not detect any S100A8/A9 secretion by neutrophils stimulated with fMLP even when cells were primed with cytochalasin B, a well-known priming agent promoting fMLP-mediated neutrophil effector responses (data not shown). This confirms that degranulation is not involved in the secretion of S100A8/A9 and S100A12 from neutrophils. C5a and fMLP induce calcium mobilisation, which in turn activates the translocation of S100A8/A9 to the plasma membrane or cytoskeleton [47, 48] which could explain the disappearance of S100A8/A9 in the cytosol reported by Hetland et al. [46], although this translocation is not sufficient to allow its secretion. Other signals are therefore necessary to induce the secretion of calgranulins. Another possible route of secretion for calgranulins is vesicular secretion which depends on intracellular membrane-bound intermediates that need to fuse with plasma membranes to release cargo into the extracellular space. Such mechanisms involve either secretory lysosomes, exosomes derived from multivesicular bodies, or microvesicle shedding from cell surfaces [49]. Since very low levels of S100A8/A9 and S100A12 are detected in the vesicular fractions from differential centrifugation (data not shown), we conclude that the vesicular secretion pathway is not the main route used by S100A proteins to reach the extracellular environment.

IL-1*β* is perhaps the most studied protein secreted through an alternative secretion pathway. Although neutrophils are weak producers of IL-1*β*, DAMPs like MSU crystals and phagocytic particles are potent inducers of IL-1*β* secretion by monocytes [50]. The same stimuli induced S100A8/A9 and S100A12 secretion by neutrophils, suggesting common mechanisms of secretion between IL-1*β* and calgranulins. Secretion of IL-1*β* necessitates inflammasome activation consecutive to decreased intracellular K^+^ concentrations and the generation of oxidative stress [34, 51]. Closure of ATP-sensitive K^+^ channels by glibenclamide or inhibition of NOX-2 activation by DPI prevented the release of both calprotectin and S100A12 from resting and stimulated neutrophils without affecting cell viability. This suggests that, as for IL-1*β*, calgranulin secretion requires ROS production and K^+^ fluxes and that secretion of calgranulins and IL-1*β* might be linked. This hypothesis is supported by the close association of high concentrations of IL-1*β* and calgranulins in the serum of patients with autoinflammatory diseases harboring mutations in pyrin or pyrin-associated proteins [38].

In summary, we have identified a number of inflammatory stimuli that induce the secretion of both S100A12 and S100A8/A9 from human neutrophils. The secretion of each protein is not intimately linked with that of the other; some agonists that induce the release of S100A8/A9 are not able to induce the exocytosis of S100A12. Therefore, different signal transduction pathways may participate in the secretion of both proteins. The identification of events leading to S100A8/A9 and S100A12 secretion might help us better understand the inflammatory conditions leading to secretion of these proteins and consequently the role of neutrophil in innate immunity and inflammation.

## Supplementary Material

ELISAs specific for S100A8, S100A9, and S100A12 homodimers, as well as S100A8/A9 heterodimer were generated using monoclonal and polyclonal antibodies produced in our laboratory. Using these antibodies, we demonstrated that most of chemotactic factors did not induce thesecretion of S100 proteins. However, MSU crystals induced the secretion of S100A8/A9 and S100A12, and this secretion was inhibited by DPI and high concentrations of K^+^.

## Figures and Tables

**Table 1:** Proteomic analysis of proteins secreted by neutrophils.

Accession number	Name	Number of peptides^a^	MSU/fMLP^b^	*P* value^c^	Fullerenes/fMLP^d^	*P* value^c^
NETosis-associated proteins						
P04040	Catalase	26	2.46	6,71*E* − 14	1,123677	4,59*E* − 05
P16401	Histone H1.5	3	1,020058	0,973432	0,745488	0,050647
Q99880	Histone H2B type 1-L	3	0,51384	0,677915	0,386307	0,237565

Granule's antimicrobial proteins and enzymes						
P14780	Metalloproteinase-9	27	1,632532	0,285746	0,757033	0,475546
P05164-3	Myeloperoxidase	46	4,544349	5,89*E* − 20	0,934431	0,071378
P08246	Neutrophil elastase	8	4,532902	0,001009	1,151382	0,1147
P02788	Lactoferroxin-C	70	5,713208	0,014768	1,489547	0,117136
O75594	Peptidoglycan recognition protein 1	6	3,00764	0,00035	0,892349	0,189002

Cytoplasmic/intracellular proteins						
P07195	Lactate dehydrogenase B chain	3	1,563257	0,274256	1,324116	0,095393
P04083	Annexin A1	6	1,199672	0,307777	0,695102	0,001041
P12429	Annexin A3	3	1,917971	0,038612	0,974026	0,701223
P26447	S100A4	3	1,885171	0,033789	1,114386	0,027142
P31949	S100A11	2	1,926023	0,159743	1,063338	0,363006
P35579	Myosin-9	45	1,276348	0,097059	1,114768	6,50*E* − 05
Q53GK6	Beta actin variant	12	1,649348	0,235332	1,09936	0,612781

Calgranulins						
P05109	S100A8	15	5,706729	1,04*E* − 06	2,008216	0,001211
P06702	S100A9	17	5,775399	1,83*E* − 05	1,982834	0,000234
P80511	S100A12	7	2,426018	0,042156	1,293581	0,001423

^a^Number of peptides identified by isobaric tags for relative and absolute quantitation.

^b^Ratio of the different proteins in supernatants from neutrophils stimulated with MSU crystals over neutrophils stimulated with fMLP (which does not induce secretion of calgranulins).

^c^ *P* value for the identification of the peptides.

^d^Ratio of the different proteins in supernatants from neutrophils stimulated with fullerenes over neutrophils stimulated with fMLP (which does not induce secretion of calgranulins).
