# Supplementary material for: Secretion of S100A8, S100A9, and S100A12 by Neutrophils Involves Reactive Oxygen Species and Potassium Efflux
Source: J Immunol Res. 2015 Dec 30;2015:296149. doi: 10.1155/2015/296149 (PMC4736198; doi:10.1155/2015/296149)
Supplement: Supplementary file 1 — ELISAs specific for S100A8, S100A9, and S100A12 homodimers, as well as S100A8/A9 heterodimer were generated using monoclonal and polyclonal antibodies produced in our laboratory. Using these antibodies, we demonstrated that most of chemotactic factors did not induce thesecretion of S100 proteins. However, MSU crystals induced the secretion of S100A8/A9 and S100A12, and this secretion was inhibited by DPI and high concentrations of K+. [file 296149.f1.pdf]

Supplementary Data

Methods

Production and purification of monoclonal and polyclonal antibodies. Human S100A8, S100A9 and S100A12 mouse monoclonal antibodies (mAb) were generated using the ClonaCell® – H hybridoma cloning kit (Stemcell Technologies) according to the manufacturer's instructions. Clones 1F8, (anti-S100A8), 6B4 (anti-S100A9) and 2A10 (anti-S100A12) were selected based on their specificity and their affinity/avidity for the appropriate targets according to ELISA analysis and confirmed by Western Blotting. Clones 1F8, 6B4 and 2A10 were then cultured in CELLine™ flasks (BD Biosciences) for Ab production. The supernatant was collected after 14 days and purified using Protein G columns as described by the manufacturer (Millipore). Purified rabbit IgGs directed against human S100A8, S100A9 and S100A12 were prepared as previously described (8). Antibodies were kept at -20°C until used.

Direct ELISA. Recombinant human S100A8, S100A9, S100A12 and purified human calprotectin (1 to 1000 ng in 100 µl) diluted in 0.1 M carbonate buffer (pH 9.6) were added to high-binding 96-well plates and incubated at 4°C overnight. The plates were washed three times with PBS/0.1% Tween and the non-specific binding sites were blocked by the addition of PBS/0.1% Tween/2%BSA for 45 min at room temperature. One hundred µl of a solution of the mAbs 1F8, 6B4, 2A10, and 27E10, as well as pAb anti-S100A8 or anti-S100A9 diluted in PBS/0.1% Tween/2%BSA to a concentration of 1 µg/ml (0.1ug/ml for 27E10) were added. After 45 min, the plates were extensively washed and the HRP-conjugated goat anti-mouse or goat anti-rabbit IgG antibody (1:10,000) dilution was added

for 45 min. The wells were washed three times, 3,3',5,5'-tetramethylbenzidine solution (TMB) substrate was added and the reaction was stopped by the addition of H_2SO_4 0.18M. The optical density was read at 450 nm.

SDS-PAGE and Western Blot. Recombinant S100 proteins (1 µg), purified calprotectin (1 µg) and crude extracts of neutrophils (10 µg) were loaded onto 15% SDS-PAGE gels under reducing (anti-S100A8 and S100A9) or non-reducing (anti-S100A12) conditions. Proteins were transferred onto PVDF membranes and non-specific binding sites were blocked by incubating the membranes in Tris-buffered saline/0.1% Tween containing 5% milk or 5% BSA (anti-S100A12) for 1 h at RT. The membranes were then incubated overnight at 4°C with solutions of mAbs (anti-S100A8-1F8, anti-S100A9-6B4 or anti-S100A12-2A10) or pAbs (anti-S100A8, anti-S100A9 or anti-S100A12) diluted in their respective blocking buffer at 1 µg/ml (anti-S100A8 and anti-S100A9) or 2 µg/ml (anti-S100A12). After extensive washes, membranes were incubated with HRP-conjugated goat anti-mouse or anti-rabbit IgG (at 1:20 000 dilution) for 1h at room temperature, washed four times and proteins were visualized by using the ECL Western blotting reagent (GE Healthcare) as described by the manufacturer. In some cases, membranes were stripped in buffer composed of β–mercaptoethanol 0.8%, SDS 2% and Tris-HCL 6.25 mM before blocking and reblotting with other antibodies.

Production of reactive oxygen species. Neutrophils (10^7 cells/ml) were incubated with 10 µM 6-carboxy-2',7'-dichlorodihydrofluorescein diacetate for 20 min at 37˚C in presence or absence of 10 µM DPI for the last 5 min. The cells were washed once in HBSS 1X

containing 1.3 mM $CaCl_2$ and 10 mM HEPES (pH7.4), then stimulated with 1.5 mg/ml MSU crystals or 1µM PMA for increasing periods of time. Fluorescence was measured using a fluorescence ELISA reader.

Supplementary data

Characterization of novel antibodies and ELISAs for S100A8, S100A9 and S100A12.

Specific monoclonal and polyclonal antibodies recognising human S100A8, S100A9 or S100A12 were raised by immunising mice and rabbits. mAbs clones 1F8 (anti-S100A8), 6B4 (anti-S100A9) and 2A10 (anti-S100A12) were selected for specificity for their respective targets by direct ELISA and western blot. As shown in Supplementary Figure 1A, mAb 1F8 (anti-S100A8) and pAb anti-S100A8 did not recognize human or mouse S100A9, S100A12, or mouse S100A8, but bound to recombinant and native human S100A8. Similarly, the anti-S100A9 mAb 6B4 detected only human S100A9 and purified calprotectin. Binding to human S100A9, and to a lower extent mouse S100A9 was observed with the pAb against human S100A9. Each of these antibodies bound to a single band of the expected molecular weight. In contrast, bands of approximately 12, 21, 60 and 100 kDa (not shown) were detected by mAb 2A10 (anti-S100A12) in neutrophil's crude extract, presumed to be the monomeric, dimeric and hexameric forms of S100A12. Specificity of the monoclonal antibodies was then confirmed by direct ELISA using increasing concentrations of recombinant mouse and human S100 proteins (Supp. Fig 1B-G). While all mAbs were specific to human S100A8, S100A9 and S100A12 (1F8, 6B4 and

2A10 respectively) and did not recognize the murine proteins, the pAbs anti-S100A8 and anti-S100A9 bound to both human and mouse proteins.

Binding of the mAbs anti-S100A8 and anti-S100A9 to the heterodimer S100A8/A9 (calprotectin) was next investigated. The mAbs 1F8 and 6B4 bound to recombinant human S100A8 and S100A9 respectively, but had low affinity to purified human calprotectin (Suppl. Fig. 1H-J). Both 1F8 and 6B4 had lower affinity for calprotectin than the anti-calprotectin mAb 27E10.

Three sandwich ELISAs (for S100A8, S100A9 and S100A12, respectively) were then developed using these specific monoclonal and polyclonal antibodies. (Suppl. Fig. 1K-N). The ELISAs detecting S100A8 or S100A9 had lower limit of detection of 3.125 ng/ml, lower limit of quantification (LLOQ) of 6.25 ng/ml and an upper limit of quantification (ULOQ) of 100 ng/ml. Sensitivity of S100A12 ELISA was 10 times higher with a lower limit of detection of 0.39 ng/m, a LLOQ of 1.56 ng/ml and an ULOQ of 25 ng/ml. A sandwich ELISA for calprotectin using the polyclonal anti-S100A9 as capture antibody and the commercially available mAb 27E10 (which recognize an epitope formed by the association of S100A8 and S100A9) as detecting antibody was also developed (calprotectin purified from neutrophils was used as standard curve). This ELISA proved highly sensitive with a lower limit of detection of 0.39 ng/ml. Specificity of the sandwich ELISAs detecting S100A8 or S100A9 was confirmed by adding increasing concentrations of purified human calprotectin (7.8 to 4 000 ng/ml). Both ELISAs showed high specificity, with almost no cross-reaction with purified calprotectin below 250 ng/ml (Suppl. Fig. 1O and P). This specificity probably result from the fact that the polyclonal and monoclonal antibodies bind to a common epitope on the C-terminal end of S100A8 or S100A9 (data

not shown), restricting the detection to the homodimers. Higher concentrations of calprotectin led to a minor detection of S100A8 and S100A9 (0.04% and 0.1% respectively) likely due to a contamination of the calprotectin preparation with S100A8 and S100A9 homodimers. Therefore, these indirect ELISAs detect specifically the respective proteins in monomer/homocomplex forms and can be used to measure specific forms present in biological fluids like serum, supernatants of stimulates cells or subcellular compartments of cells.

Figure S1. Specificity of monoclonal and polyclonal antibodies against human S100A8, S100A9, and S100A12 proteins. A) mAbs and pAbs against S100A8, S100A9, and S100A12 specifically bind to their respective target antigens. Recombinant human S100A8, S100A9, S100A12, mS100A8 (murine S100A8), mS100A9 (murine S100A9), purified human calprotectin, and neutrophils crude extract were loaded onto SDS-PAGE and detected by western blotting using our monoclonal (1F8 (anti-S100A8), 6B4 (anti-S100A9), 2A10 (anti-S100A12)) and polyclonal antibodies as noted. B to G) Recombinant human and murine S100A proteins (1 to 1000 ng in 100 µl) and purified human calprotectin were loaded in high-binding 96-well plates to perform standard ELISA with B) 1F8 mAb, C) rabbit pAb against S100A8, D) 6B4 mAb, E) rabbit polyclonal against S100A9, F) 2A10 mAb, and G rabbit pAb against S100A12 as described in materials and methods.

Figure S2. Comparison of binding activity of the different antibodies on wells coated with A) S100A8, B) S100A9, and C) S100A8/A9.

Figure S3. Calibration curves of ELISAs specific for calganulins. Monoclonal or polyclonal Abs were coated in high-binding 96-well plates to perform sandwiches ELISA for A) S100A8, B) S100A9, C) S100A8/A9, and D) S100A12. E and F) Absence of cross-reactivity of S100A8 and S100A9 ELISAs. Purified human calprotectin (7.8 to 4000 ng/ml) were loaded into the well precoated with E) 1F8 or F) 6B4 to perform S100A8 and S100A9 sandwich ELISA and verify the absence of detection by the polyclonal Abs.

Figure S4. Limited secretion of S100A8/A9 and S100A12 induced by chemoattractants. Neutrophils were stimulated with C5a, IL-8, LTB4, PAF and fMLP, or their diluants for 1 hour. A) S100A8/A9 and B) S100A12 were then quantified in the supernatants. Data are from 5 experiments performed on different blood donors.

Figure S5. DPI inhibits the production of reactive oxygen species by neutrophils induced by MSU crystals and PMA. Neutrophils were preincubated with 10 µM DPI for 5 min before being stimulated with A) 1.5 mg/ml MSU crystals or their diluent (HBSS), or B) 1 µM PMA or its diluent (DMSO). Data is from one experiment representative of 3 experiments performed on cells from different blood donors.

Figure S6. High extracellular K^+ concentrations inhibit the secretion of S100A8/A9 induced by MSU crystals. Neutrophils were stimulated with MSU crystals or its diluent (HBSS) in presence or absence of 130 mM K^+ for 1h at 37°C. S100A8/A9 in the supernatants was quantified by ELISA. Data are the mean ± sem of 3 experiments performed on cells from different donors.

Figure S7. Cytochalasin B does not increase the secretion of S100A8/A9. Neutrophils were stimulated with 10^{-7} M fMLP or its diluent (DMSO) in presence or absence of 10 µM cytochalasin B for 1h at 37˚C. S100A8/A9 in the supernatants was quantified by ELISA. Data are the mean ± sem of 3 experiments performed on cells from different donors.

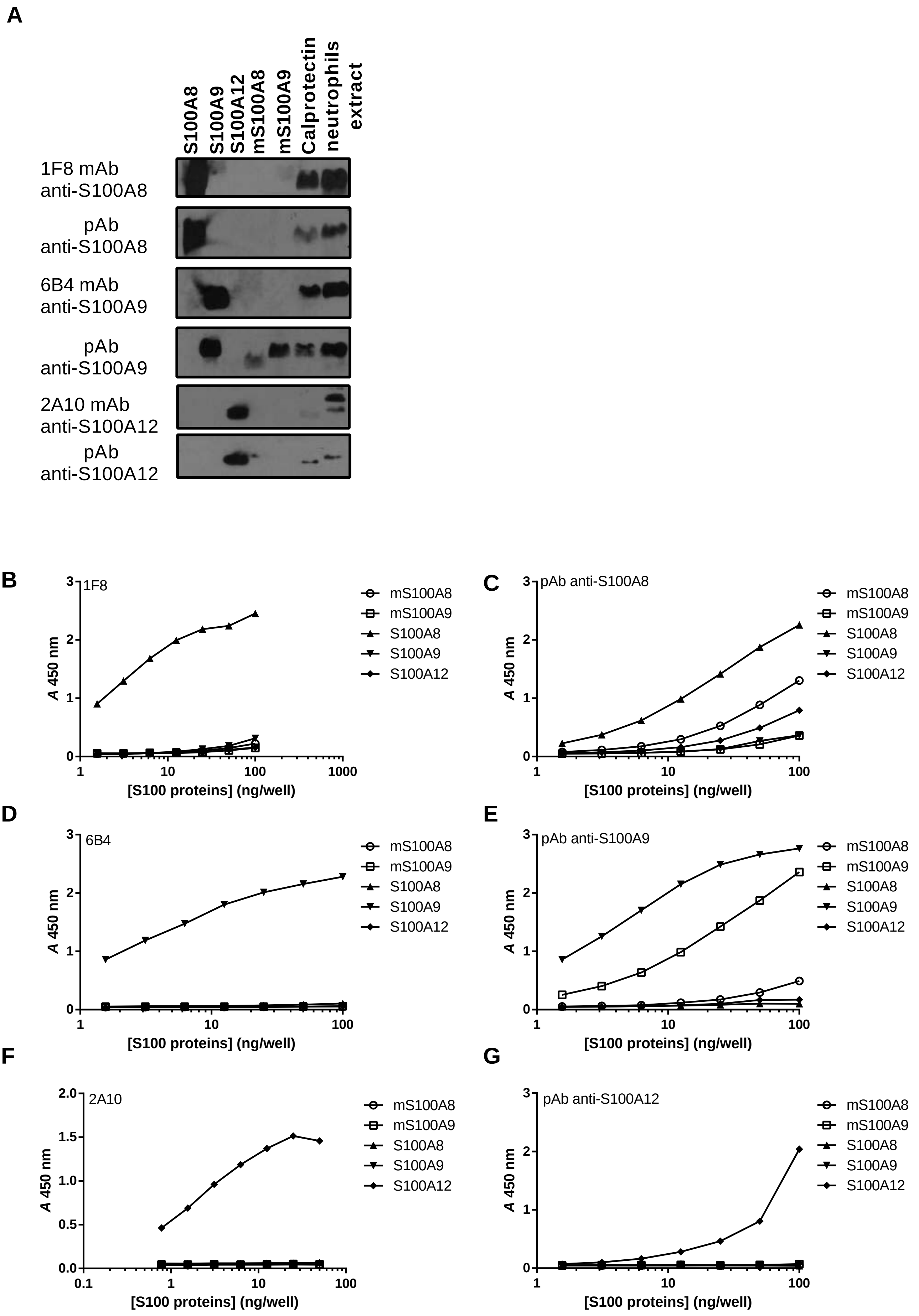

Supplementary Figure 1

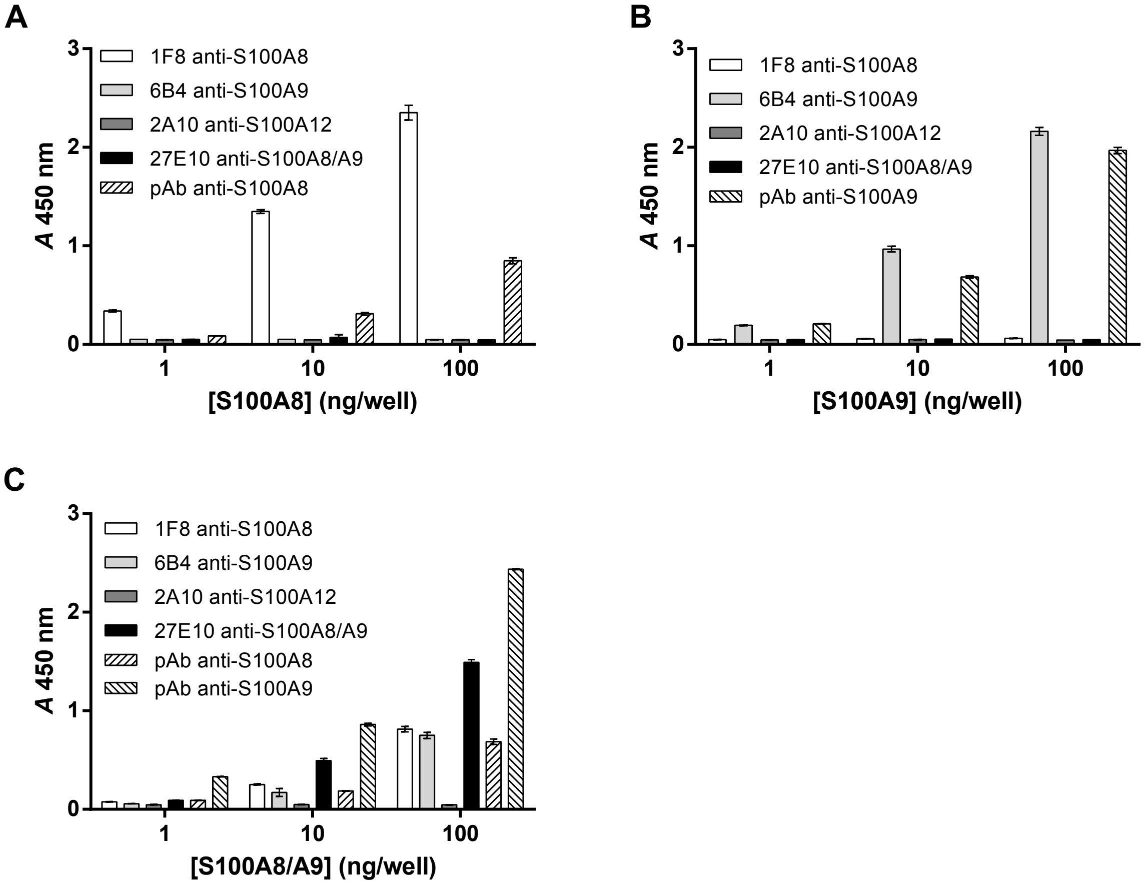

Supplementary Figure 2

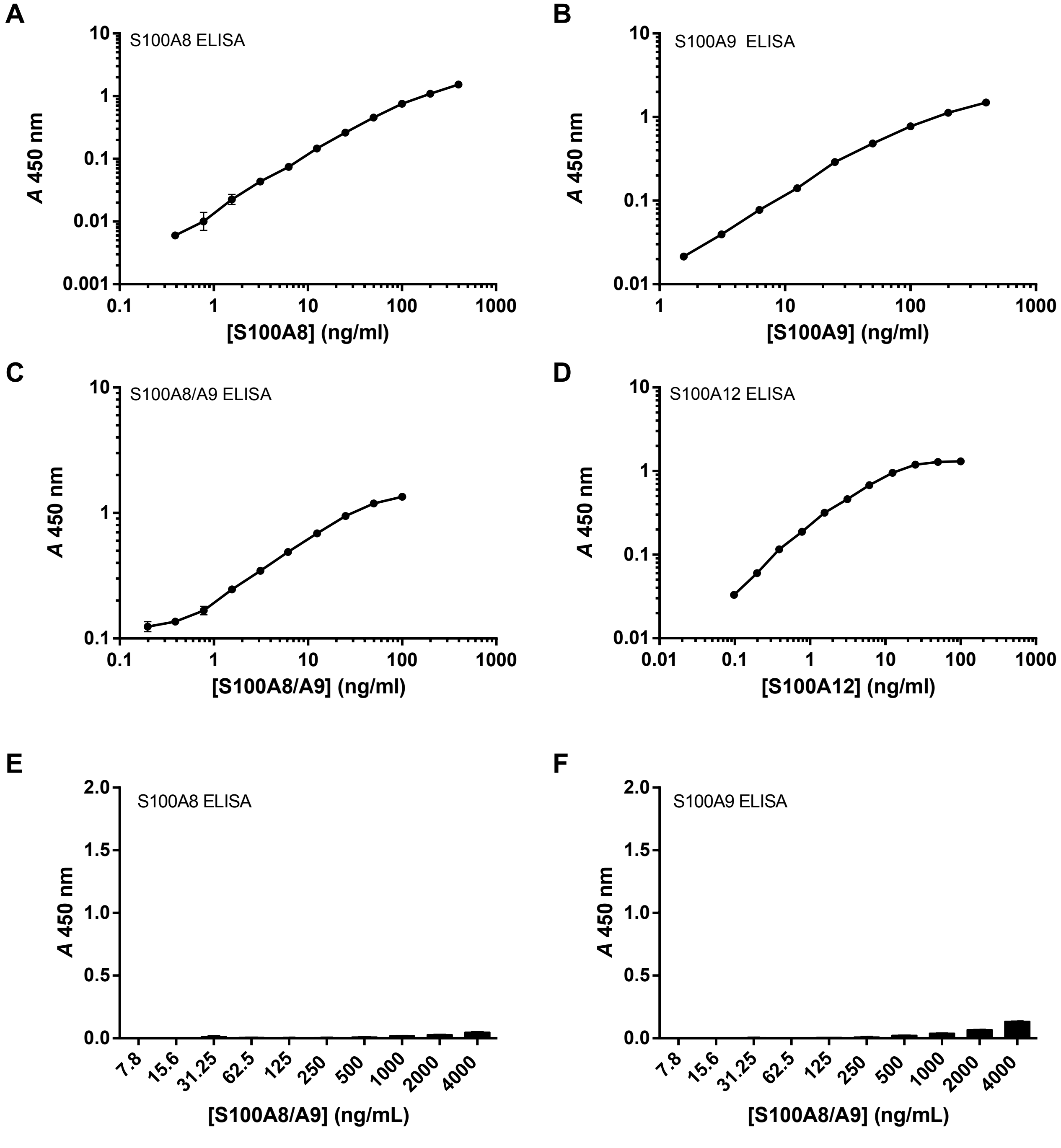

Supplementary Figure 3

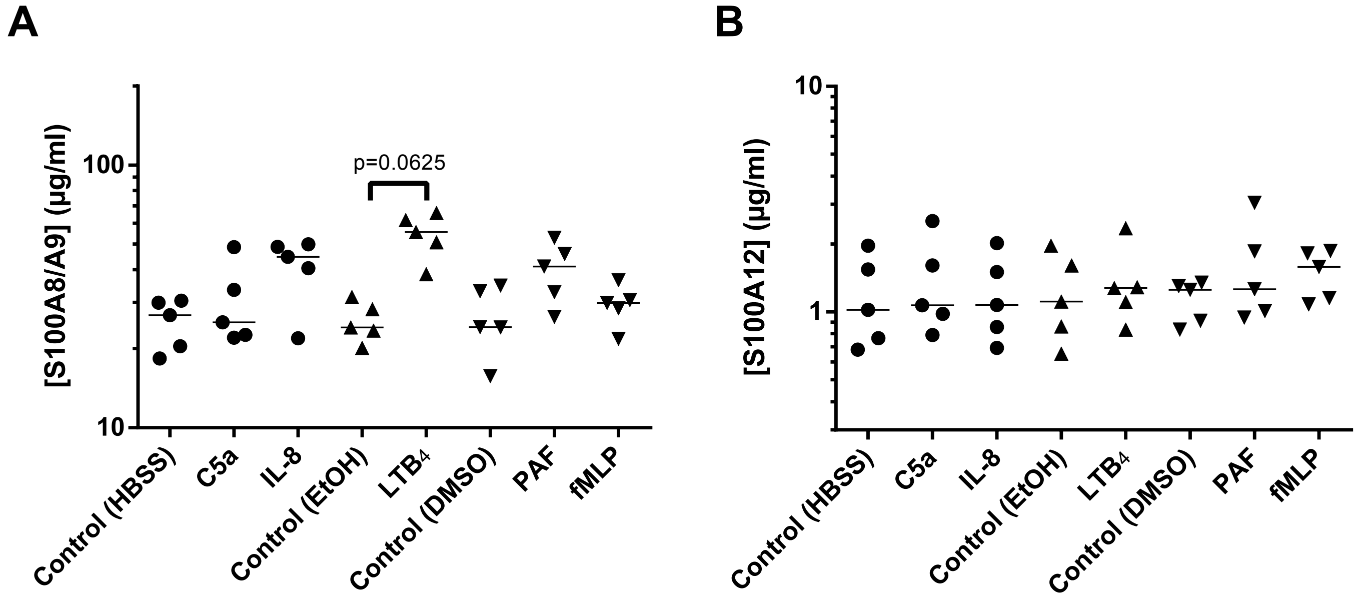

Supplementary Figure 4

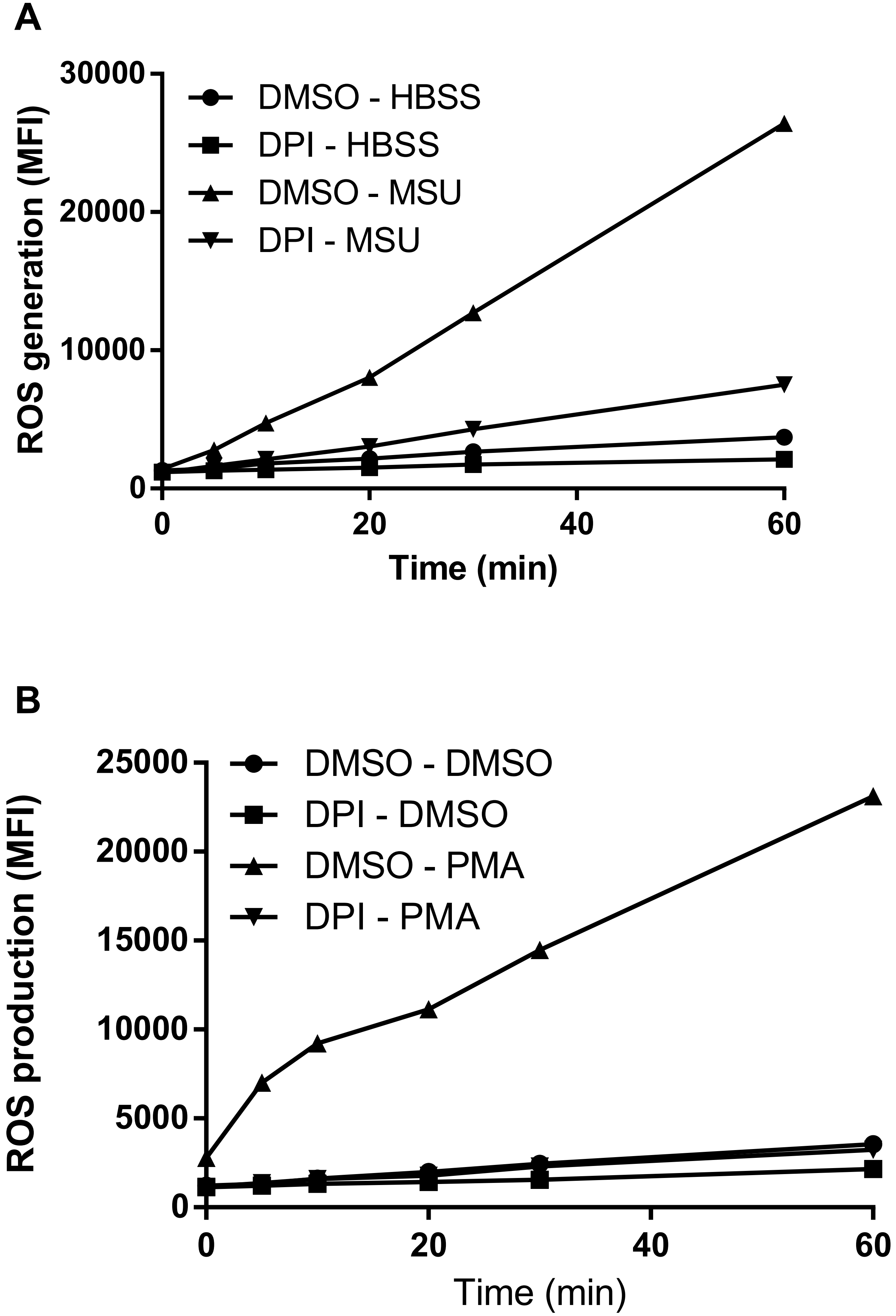

Supplementary Figure 5

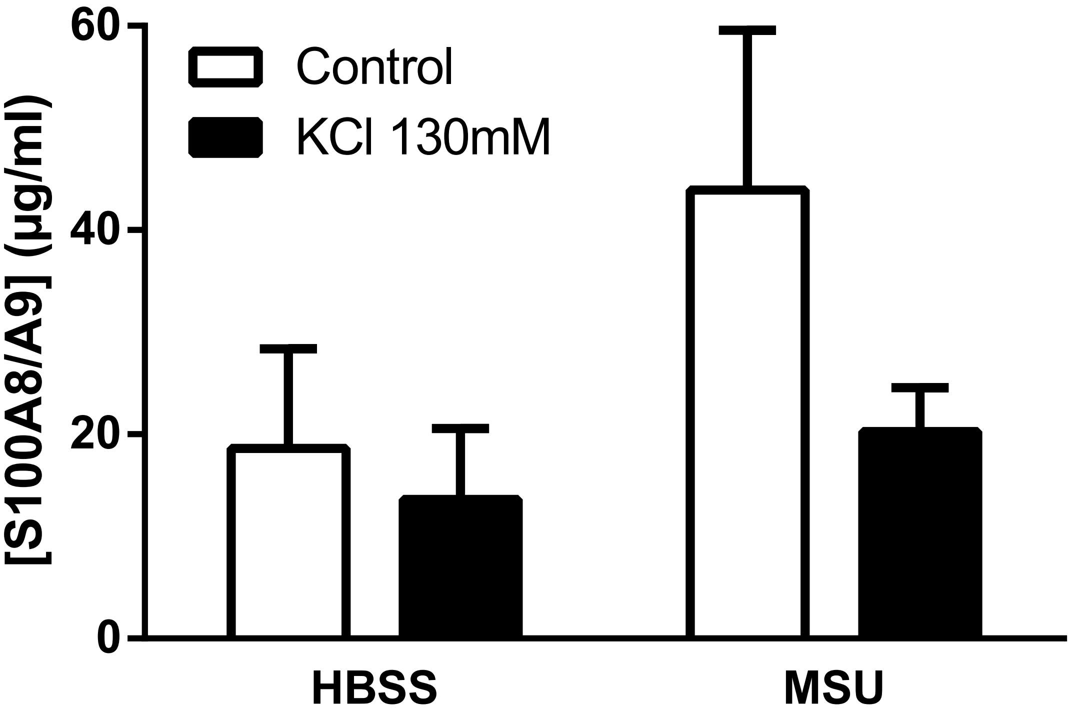

Supplementary Figure 6

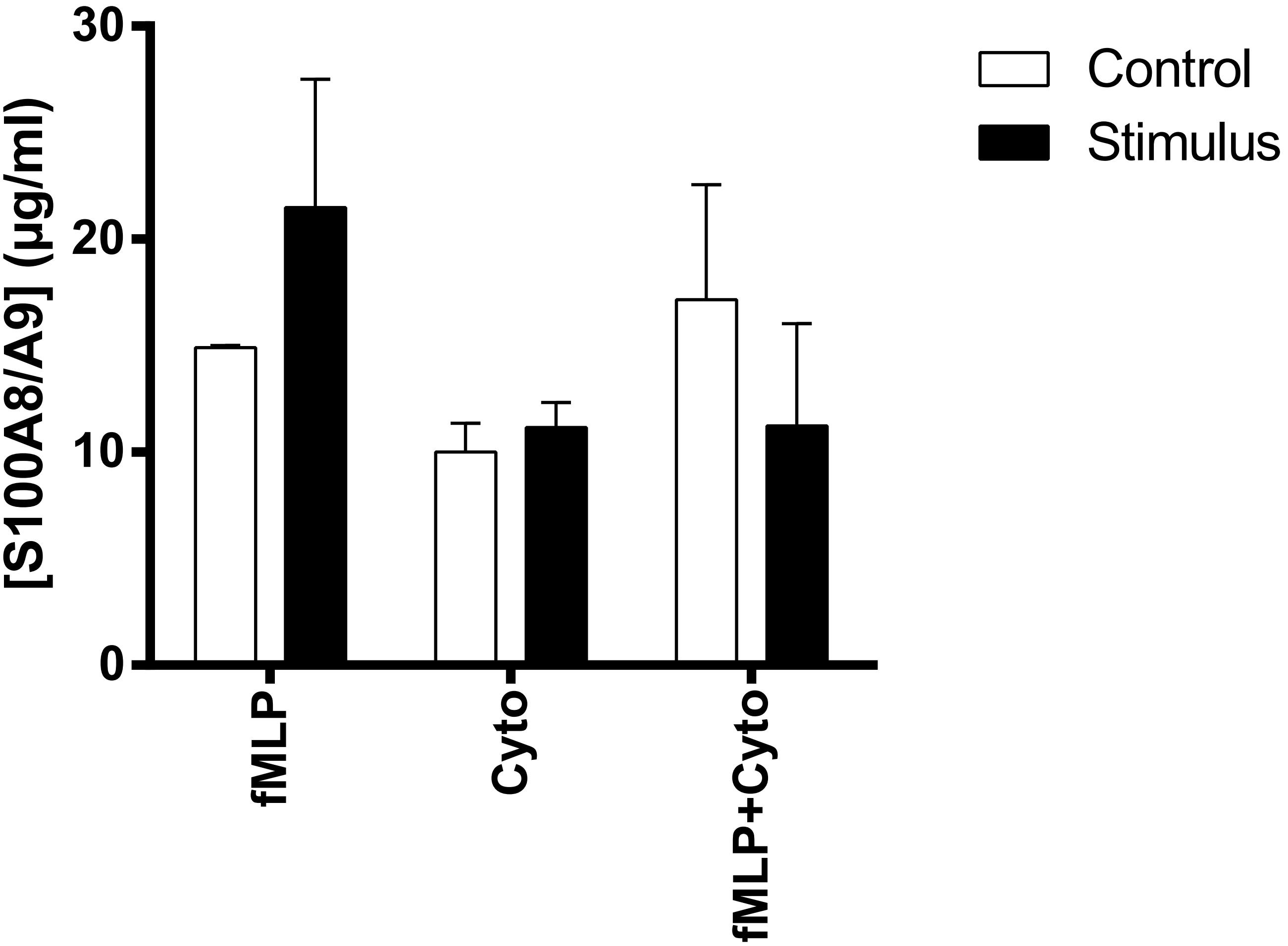

Supplementary Figure 7
